# Parenting Knowledge, Parental Investments, and Early Childhood Development in Rural Households in Western China

**DOI:** 10.3390/ijerph17082792

**Published:** 2020-04-17

**Authors:** Jingdong Zhong, Yang He, Jingjing Gao, Tianyi Wang, Renfu Luo

**Affiliations:** 1 School of Economics, Peking University, Beijing 100871, China; jdzhong@pku.edu.cn; 2 China Center for Agricultural Policy, School of Advanced Agricultural Sciences, Peking University, Beijing 100871, China; heyang.ivy@pku.edu.cn (Y.H.); gaojj@pku.edu.cn (J.G.); tianyi_ccap@pku.edu.cn (T.W.)

**Keywords:** parenting knowledge, parental investments, early childhood development, human capital, rural China

## Abstract

This paper investigates the relationships between caregivers’ parenting knowledge and early childhood development, based on a survey conducted in 1715 rural households in 100 villages located in an undeveloped rural area of western China. The results find that, first, caregivers’ parenting knowledge is positively and significantly associated with children’s development outcomes, including cognitive, language, motor, and social–emotional development; second, caregivers’ parental investments significantly mediate the link between parenting knowledge and early childhood development; third, in contrast with other parental investments, play materials (in terms of variety and quantity) and play activities in the households are the strongest mediators. Our findings might be informative for policy makers to design policies targeted to foster human capital formation in rural China.

## 1. Introduction

Early childhood development (ECD) remains a significant issue in rural China. New estimates show that a large proportion (85%) of the children aged 0–3 years in four major subpopulations of rural China have not reached their full developmental potential in at least one kind of outcome [1]. Specifically, 49%, 52%, 30%, and 53% of these children exhibit cognitive, language, motor, and social–emotional delays, respectively [1]. As ECD outcomes are vital to welfare in adulthood, such as health status [2,3], labor market returns [4,5], social mobility [6], and other adult outcomes [7,8], early developmental delays are detrimental to human capital formation and long-term economic growth in China [9].

Previous studies in China have found that parental investments are poor in rural areas. Parental investments are parental expenditures on material and time that benefit offspring [10]. In terms of material investments, rural households usually own less play materials, both in quantity and variety [11,12]. In terms of time investments, only 12.6% of caregivers in rural households read with their child, and less than 40% play and sing songs with their children [13]. Similarly, only 13.8% of rural caregivers tell stories to their child [14]. Additionally, rural children play alone for over 2.5 hours per day on average, which indicates an absence of interactive parenting activities between these children and their caregivers [15]. So far, there are some parenting intervention programs in place in some areas of rural China, such as Yunnan, Hebei Province [16], and Shaanxi Province [17]. These programs find that the interventions lead to the improvement in child’s cognitive development by 0.23–0.27 standard deviation (SD), which is accompanied with the increase in parental investments by 0.35–0.72 SD. That is, parental investments might be positively associated with child’s cognitive development.

Poor parental investments are usually accompanied by poor parenting knowledge. Parenting knowledge is parental knowledge of child development [18]. Parenting knowledge has been shown to be important to ECD outcomes, as it helps caregivers to create a stimulating home environment in which to interact with their children [18,19,20]. However, at least half of caregivers in rural areas do not know how to successfully invest in their children to stimulate their development [13,19,20,21]. This indicates that poor parenting knowledge may be one of the main drivers for poor parental investments, and in turn, may explain the low ECD levels in rural China. To the best of our knowledge, however, there is no population-wide public data on children and caregivers during early childhood in rural China, and few studies have explored the mediating effects of parental investments in the relationships between caregivers’ parenting knowledge and children’s development outcomes in rural China.

This paper has the following three specific objectives. First, it aims to investigate the relationships between parenting knowledge and early childhood development in Chinese rural households. Second, it aims to figure out whether parental investment works as a mediator in the link between them. Third, due to the fact that different parental investments have varied predictive abilities on different development outcomes [11,12], it also plans to further identify the mediating effects of different parental investments on the relationships between parenting knowledge and different development outcomes.

## 2. Methods

### 2.1. Sampling

The survey was conducted in 22 nationally-designated poverty-stricken counties in a northwestern province of China, which is a typical undeveloped rural area. In 2016, the per capita income in this province ranked in the bottom half among all provinces, and more than 40% of the population in this province lived in rural areas.

We followed the three-step protocol to select the study sample. First, as part of large-scale randomized controlled trial, a list of all 245 towns was obtained in 22 sample counties by excluding the township in each county that housed the county seat. Second, according to the sample size calculation for the large-scale randomized controlled trial, 100 towns were randomly chosen from the sampling frame using a computerized random number generator. Within each town, one village was randomly selected to participate in the survey. Finally, based on the list of registered births from the local official in each sample village, all children aged 6–24 months and their caregivers were enrolled in the survey. The reason why we chose children in this age range is that the parenting services would last for a year in the large-scale randomized controlled trial. Since most of the children will begin their life in kindergarten after 36 months of age, children in the current sample frame should be younger than 24 months of age. No household refused participation. Our final sample included a total of 1715 households from 100 villages.

### 2.2. Ethical Approval

All subjects gave their informed consent for inclusion before they participated in the study. The study was conducted in accordance with the Declaration of Helsinki, and the protocol was approved by the Ethics Committee of Stanford University, Stanford, CA, USA (Number: 35921).

### 2.3. Measures

In 2016, we collected data on four types of information from all sample households: child’s development outcomes, caregivers’ parenting knowledge, caregivers’ parental investments, and socioeconomic status. We used the following instruments in the survey.

(1) Bayley Scales of Infant Development version III (BSID-III). The BSID-III test was designed by [22] as a standardized instrument to evaluate the developmental functioning of a child from birth to age three [23]. The test takes into account each child’s age in months and whether he or she was premature at birth. These two factors are combined with the child’s performance on a series of tasks. It includes four scales to assess a child’s cognitive, language, motor, and social–emotional development, respectively. These scales have been formally adapted to the Chinese environment and have been used in multiple studies across rural China [1]. The assessments of cognitive, language, and motor skills depend on scores that are given according to a child’s successful completion of the tasks, for example, “calms down when being picked up” (cognitive scale), “regards person momentarily” (language scale), “hands are fisted” (motor scale) [22]. The social–emotional score comes from caregivers’ responses to relevant questions developed from the Greenspan Social–Emotional Growth Chart, for example, “takes a calm and enjoyable interest in most sounds” (social-emotional scale) [24]. Higher scores indicate better development of the child.

The sample child was administered the BSID-III test by trained enumerators using a standardized set of toys and a detailed scoring sheet. The caregiver was present but was not allowed to help the child during the test. All enumerators attended a week-long training course on how to administer the BSID-III before the survey, but they were blind to the study. Average reliability coefficients are all above 0.8 for the BSID-III scales, which demonstrates that the scales can accurately measure child development [25].

(2) Knowledge of Infant Development Inventory-P (KIDI-P). Knowledge of Infant Development Inventory (KIDI) was designed by [26] and has been widely used in international studies [27,28]. The KIDI-P is a subsection of KIDI and has been formally adapted to the Chinese language and context, i.e., it has been translated into Chinese [20]. The KIDI-P includes two parts: one is the knowledge test to evaluate the caregivers’ knowledge of child development, and the other is the belief test to access a caregiver’s beliefs on child achievements at a certain age.

The primary caregiver of each child was identified as the individual most responsible for the child’s daily care. The primary caregiver was then administered a detailed questionnaire adapted from the knowledge test of the KIDI-P to access their parenting knowledge. The questionnaire consists of 39 items concerning children’s normative behaviors, for example, “when toddlers are strongly attached (bonded) to their parents, they are clingier and tend to stick close to mom or dad”. Caregivers were asked to use the three-point scale (agree, disagree, or unsure) to respond to each item. Each correct response was scored as positive one point, while each incorrect or unsure response was scored as no point for that item. The Cronbach’s alpha coefficients of the inventory were larger than 0.8, indicating that the internal consistency is good in our sample [25]. The total score of the knowledge test was calculated as the percentage of correct-answered questions. A high score indicates better parenting knowledge.

(3) Family Care Indicators (FCI). The FCI was developed by the UNICEF experts with preliminary piloting to measure parental investments [29]. Previous studies have demonstrated that the FCI yields both reliability and validity in developing countries [30]. The FCI has also been adapted to the Chinese language and context, i.e., translated into Chinese, in previous studies across rural China [11,12].

The primary caregiver was administered a detailed questionnaire adapted from the FCI. The items in the FCI are grouped into seven subgroups. The items in three of the subgroups (source of play materials, variety of play materials, and play activities) were scored as yes = 1 and no = 0 (presence or absence of play material or activity). The items in the remaining four subgroups (household books, picture books, play materials, magazine) were scored by the real number of the relevant materials in the household. The Cronbach’s alpha coefficients of the FCI inventory were larger than 0.8, indicating that the internal consistency is good in our sample [25]. The total scores of FCI were calculated by summing up the scores of the relevant items. A higher total score of FCI indicates more parental investments.

(4) Socioeconomic survey. The questionnaire was administered to the primary caregivers to collect data on the socioeconomic characteristics of each household, including the child’s gender, the child’s age in months, whether the child is born with low birthweight, the caregiver’s age, the caregiver’s completed years of schooling, and whether the mother is the child’s primary caregiver.

### 2.4. Participants

In terms of socioeconomic characteristics of participants, just over half (52%) of the children were male; the children were slightly over 14 months old on average; 4% of the children were born with low weight; the primary caregivers were around 35 years old on average; the caregiver’s number of years of schooling was approximately eight on average; and the mother was the primary caregiver in 70% of the households. In rural areas, children are taken care of by their grandmothers when their mothers migrate out to large cities for higher income [31].

### 2.5. Statistical Analysis

In order to examine the relationship between the caregiver’s parenting knowledge and their child’s development outcomes, we used the multivariate regression. The dependent variable is the child’s cognitive, language, motor, and social–emotional score in the BSID-III test. The independent variable is the caregiver’s total score of knowledge test in the KIDI-P. Covariates are socioeconomic characteristics, including the child’s gender, the child’s age in months, whether the child was born with low birthweight, the caregiver’s age, the caregiver’s number of years of schooling, and whether the mother is the child’s primary caregiver. The village fixed effects (FE) are used to control for the unobserved heterogeneity at the village level. We used the ordinary least squares (OLS) estimates to estimate the regression. We adjusted the standard errors to account for clustering at the village level.

Furthermore, to identify the mediating role of parental investments in the relationship between parenting knowledge and development outcomes, we used the mediation model. The mediator is the caregiver’s FCI total score, and the other variables are the same as above. Following [32], standard errors (S.E.) of the indirect effects were computed by using the bootstrap method based on resampling with 1000 replications. Three types of 95% confidence interval (CI), i.e., percentile interval, bias-corrected (BC) interval, and bias-corrected and accelerated (BCa) interval, were also calculated to test the statistical significance of the indirect effects. The percentile 95% CI uses usual sampling distribution cutoffs without bias correction, while the BC 95% CI corrects for a bias in the distribution of bootstrap estimates of standard errors. The BCa 95% CI corrects for bias and skewness in the distribution. If the CI does not contain zero, the indirect effect should be considered statistically significant.

Given the multi-factorial measure of the parental investments, we then examined which subscale is strong mediator. We used the FCI seven subscale scores to replace the FCI total score as mediators and reported the estimates of indirect effects of parenting knowledge on child’s cognitive, language, motor, and social–emotional development through these mediators, respectively. Stata 15.0 (StataCorp LLC, College Station, TX, USA) was used for data analysis.

## 3. Results

### 3.1. Descriptive Statistics on Parenting Knowledge and Development Outcomes

The mean of children’s cognitive, language, motor, and social–emotional scores in our sample are 96.00, 92.41, 97.21, and 85.94, respectively. In terms of parenting knowledge, the average parenting knowledge score of sample caregivers is 0.52. That is, the caregivers in our sample only correctly answered 52% of questions in the questionnaire. 

### 3.2. Relationship Between Parenting Knowledge and Development Outcomes

Table 1 reports the OLS estimates of correlation between parenting knowledge and development outcomes. The caregiver’s parenting knowledge is positively and significantly associated with their child’s cognitive, language, motor, and social–emotional development at the 1% significance level. In terms of effect size, one SD increase in the caregiver’s parenting knowledge score is accompanied by a 0.15 SD, 0.18 SD, 0.11 SD, and 0.17 SD increase in their child’s cognitive, language, motor, and social–emotional score on average. The results in Table 1 suggest that interventions aimed at improving caregivers’ parenting knowledge might be effective to foster ECD outcomes in rural households in western China.

### 3.3. Mediation Effects of Parental Investments 

Table 2 reports the OLS estimates of the correlations between the parenting knowledge, parental investments, and development outcomes. As shown in columns (1)–(4), after controlling for parental investments, the correlations between parenting knowledge and development outcomes are still significantly positive at the 1% level, and the effect sizes are smaller than the estimates reported in Table 1. Additionally, parental investments are positively and significantly associated with children’s cognitive, language, motor, and social–emotional development at the 1% significance level. A one SD increase in the caregiver’s FCI score is accompanied by a 0.11 SD, 0.10 SD, 0.12 SD, and 0.15 SD increase in their child’s cognitive, language, motor, and social–emotional score. As shown in Column (5), parenting knowledge is significantly associated with parental investments at the 1% level, with a one SD increase in the parenting knowledge score corresponding to a 0.17 SD increase in the FCI score on average. The results in Table 2 indicate that mediating effects of parental investments might exist in the link between caregivers’ parenting knowledge and development outcomes.

As shown in Table 3, for the estimated indirect effects of the caregiver’s parenting knowledge on their child’s cognitive, language, motor, and social–emotional development through parental investments, the point estimates are significantly larger than zero, and the three 95% CIs do not contain zero (columns (3)–(5)), which strongly suggests that the indirect effects through parental investments are statistically significant.

In terms of cognitive development (Table 4), we find that the variety and the number of play materials are the strongest mediators, through both of which a one SD increase in the caregiver’s parenting knowledge is associated with a 0.02 SD increase in their child’s cognitive score at the 1% significance level. In terms of language development (Table 5), the variety of play materials, the play activities, and the number of play materials are three strong mediators, with indirect effects of 0.04 SD, 0.03 SD, and 0.03 SD at the 1% level, respectively, followed by the number of books. Similarly, in terms of motor development (Table 6), the variety of play materials, the play activities, and the number of play materials are also strong mediators, with indirect effects of 0.06 SD, 0.03 SD, and 0.02 SD at the 1% level, followed by the sources of the play materials, the number of picture books, and the number of books. In terms of social–emotional development (Table 7), the variety of play materials, the play activities, the number of books, and the number of play materials are significant mediators, while the other subscales are not statistically significant.

In short, different parental investments play different roles in the relationships between parenting knowledge and early childhood development. Compared with the other subscales of FCI, however, the variety of play materials, the play activities, and the number of play materials are the strongest mediators. That is, caregivers with a higher level of parenting knowledge tend to invest more in play materials (in terms of variety and quantity) for their children and engage in more play activities with them, which in turn improves development outcomes.

## 4. Discussion

In this paper, we aimed to measure caregivers’ parenting knowledge and children’s early development outcomes in rural China, and explore their relationships. This paper has three main findings, as follows. First and foremost, after socioeconomic characteristics and village heterogeneity are controlled, caregivers’ parenting knowledge is positively and significantly associated with children’s neurodevelopment, including cognitive, language, motor, and social–emotional development. Second, parental investment is a significant channel through which caregivers’ parenting knowledge is linked to children’s development outcomes. Last but not least, different parental investments play different roles between parenting knowledge and development outcomes. In contrast with other parental investments, the variety of play materials, the play activities, and the number of play materials in the households are the strongest mediators. 

In a healthy children population, the mean scores (standard deviation) are expected to be 105 (9.6), 109 (12.3), 107 (14), and 100 (15) for the cognitive scale [33], the language scale [34], the motor scale [35], and the social–emotional scale [22], respectively. That is, the mean of children’s cognitive, language, social–emotional scores in our sample are about one deviation lower than the reference means of the healthy population. In terms of parenting knowledge, the average parenting knowledge score of sample caregivers is much lower than not only the expected average score (0.72) [26], but also the average score (0.81) of sample mothers in three low-income counties in the United States [36]. The comparisons indicate a relatively low level of parenting knowledge among caregivers in our sample.

Given that children’s early skills development would exert impacts on their lifelong welfare [2,3,4,5,6,7,8], as well as on the country’s sustainable economic growth and development in the long term [9], the findings of this paper show that caregivers’ parenting knowledge during early stages is indeed of great importance. In rural China, the relatively low level of parenting knowledge among caregivers would inevitably restrain the development of their children, and might partly explain the severe developmental delays of children in these areas. 

Furthermore, the findings also suggest the key role of parental investment in the relationships between parenting knowledge and ECD outcomes. That is, caregivers who have a higher level of parenting knowledge put more parental investments in their children, which corresponds to better development outcomes of the children. This is consistent with existing studies that have emphasized the importance of parental investments in children’s early development [37,38].

On the one hand, in Chinese rural households, material investments in children, like play materials, have been found to be very few, not only in variety and but also in quantity, which, in turn, is harmful to children’s development outcomes [11,12]. The findings in this paper show that, in contrast with their counterparts with lower parenting knowledge, caregivers with higher parenting knowledge invest in more play materials (in terms of both variety and number of play materials) in the households. This increase in material investments builds a strong bridge between higher parenting knowledge and better development outcomes.

On the other hand, previous studies have documented that caregivers’ time investments in stimulating activities are productive inputs for the cognitive skill development of children, both in developed countries such as Australia [39] and the United Kingdom [40], as well as in rural areas of China [13,14,15]. Our findings further suggest that, besides children’s cognitive development, caregivers’ parental investments are also positively and significantly associated with children’s language, motor, and social–emotional development in rural households. In addition to the material investments discussed above, caregivers with higher parenting knowledge also engage in significantly more parenting activities. That is, time investments are important in the relationships between parenting knowledge and development outcomes. These findings add to the burgeoning ECD play-based learning literature around the world, which emphasizes the importance of play-based learning on early childhood development [41].

We acknowledge that our study faces some limitations. First, our sample was only collected from a typical rural area in western China, so the conclusions cannot be simply generalized to other contexts. Second, the findings based on the OLS estimates do not indicate causal inference, although it is helpful to understand the relationships of parenting knowledge, parental investments, and child development outcomes. Further investigation is needed to explore the causal effects of parenting knowledge on parental investments and early childhood development. 

## 5. Conclusions

Despite these limitations, the findings of this study still have important implications. Considering the strong correlation between parenting knowledge and development outcomes, targeted interventions to increase the level of caregivers’ parenting knowledge, especially aimed at increasing play materials (in terms of variety and quantity) and play activities, might be effective to improve human capital development in rural areas. The findings in this paper indicate that improvement in caregivers’ parental investments might be an important channel between parenting knowledge and early development outcomes, and thus deserves much attention in the analysis. We believe that the findings here are informative for the policy-makers to design targeted policies to boost early childhood development in rural areas.

## Figures and Tables

**Table 1:** Correlation between parenting knowledge and development outcomes.

Independent Variables	Cognition	Language	Motor	Social-Emotion
	(1)	(2)	(3)	(4)
Knowledge	0.15 *** (0.04)	0.18 *** (0.04)	0.11 *** (0.03)	0.17 *** (0.04)
Male	−0.08 (0.05)	−0.24 *** (0.05)	−0.04 (0.04)	−0.01 (0.05)
Month	0.004 (0.005)	0.04 *** (0.005)	0.10 *** (0.005)	0.01 * (0.004)
Low birth weight	−0.39 *** (0.15)	−0.30 ** (0.13)	−0.22 ** (0.11)	−0.28 * (0.15)
Age of Caregiver	−0.003 (0.004)	−0.002 (0.004)	−0.004 (0.004)	0.004 (0.004)
Education of Caregiver	0.02 ** (0.01)	0.04*** (0.01)	0.03 *** (0.01)	0.01 (0.01)
Mother is the child’s primary caregiver	0.28 ** (0.11)	0.13 (0.10)	0.24 *** (0.09)	0.13 (0.11)
Village fixed effects (FE)	Yes	Yes	Yes	Yes
Observations	1715	1715	1715	1715
R^2^	0.19	0.23	0.41	0.19

Notes: (i) Standardized coefficients are reported in the table, and robust standard errors presented in parentheses are clustered at the village level. (ii) *** *p* < 0.01; ** *p* < 0.05; * *p* < 0.1.

**Table 2:** Correlations between parenting knowledge, parental investments, and development outcomes.

Independent Variables	Cognition	Language	Motor	Social-Emotion	Investment
	(1)	(2)	(3)	(4)	(5)
Knowledge	0.13 *** (0.04)	0.14 *** (0.04)	0.10 *** (0.03)	0.15 *** (0.04)	0.17 *** (0.04)
Investment	0.11 *** (0.03)	0.10 *** (0.02)	0.12 *** (0.02)	0.15 *** (0.03)	
Male	−0.07 (0.05)	−0.23 *** (0.05)	−0.04 (0.04)	−0.002 (0.06)	−0.05 (0.05)
Month	−0.003 (0.005)	0.03 *** (0.004)	0.09 *** (0.005)	0.003 (0.004)	0.03 *** (0.005)
Low birth weight	−0.38 *** (0.15)	−0.29 ** (0.14)	−0.20 * (0.11)	−0.26 * (0.15)	−0.12 (0.13)
Age of Caregiver	−0.004 (0.004)	−0.002 (0.004)	−0.004 (0.004)	0.004 (0.004)	0.002 (0.004)
Education of Caregiver	0.01 (0.01)	0.03 *** (0.01)	0.02 ** (0.01)	−0.005 (0.009)	0.08 *** (0.008)
Mother is the child’s primary caregiver	0.30 *** (0.11)	0.13 (0.11)	0.26 *** (0.09)	0.14 (0.11)	0.18 (0.11)
Village FE	Yes	Yes	Yes	Yes	Yes
Observations	1715	1715	1715	1715	1715
R^2^	0.20	0.24	0.42	0.20	0.25

Notes: (i) Standardized coefficients are reported in the table, and robust standard errors presented in parentheses are clustered at the village level. (ii) *** *p* < 0.01; ** *p* < 0.05; * *p* < 0.1.

**Table 3:** Estimates of the indirect effects of parenting knowledge on development outcomes through parental investments.

Indirect Effect	Point Estimate	Bootstrap S. E.	95% CI (Percentile)	95% CI (BC)	95% CI (BCa)
	(1)	(2)	(3)	(4)	(5)
Knowledge on cognition through investment	0.02 ***	0.006	(0.008, 0.03)	(0.009, 0.03)	(0.009, 0.03)
Knowledge on language through investment	0.02 ***	0.006	(0.007, 0.03)	(0.007, 0.03)	(0.007, 0.03)
Knowledge on motor through investment	0.02 ***	0.006	(0.008, 0.03)	(0.008, 0.03)	(0.008, 0.03)
Knowledge on social-emotion through investment	0.03 ***	0.007	(0.02, 0.04)	(0.02, 0.04)	(0.02, 0.04)

Notes: (i) Bootstrap standard errors reported in column (2) are based on resampling with 1000 replications. (ii) The percentile 95% CI in column (3) uses usual sampling distribution cutoffs without bias correction; the BC 95% CI in column (4) corrects for a bias in the distribution of bootstrap estimates of standard errors; and the BCa 95% CI in column (5) corrects for bias and skewness in the distribution of bootstrap estimates of standard errors. (iii) *** *p* < 0.01.

**Table 4:** Estimates of indirect effects of parenting knowledge on cognitive development through different parental investments.

Indirect Effect	Point Estimate	Bootstrap S. E.	95% CI (Percentile)	95% CI (BC)	95% CI (BCa)
	(1)	(2)	(3)	(4)	(5)
Knowledge on cognition through source	0.003	0.003	(−0.001, 0.009)	(−0.001, 0.01)	(−0.001, 0.01)
Knowledge on cognition through variety	0.02 ***	0.007	(0.01, 0.04)	(0.01, 0.04)	(0.01, 0.04)
Knowledge on cognition through activity	0.005	0.004	(−0.001, 0.13)	(−0.001, 0.15)	(−0.001, 0.15)
Knowledge on cognition through book	0.006 *	0.003	(−0.0002, 0.14)	(0.001, 0.15)	(0.001, 0.15)
Knowledge on cognition through pbook	0.003	0.003	(−0.001, 0.009)	(−0.002, 0.008)	(−0.002, 0.008)
Knowledge on cognition through toy	0.02 ***	0.008	(0.009, 0.04)	(0.009, 0.04)	(0.009, 0.04)
Knowledge on cognition through magz	0.004	0.003	(−0.001, 0.009)	(0.0004, 0.01)	(0.0004, 0.01)

Notes: (i) The mediators are the Family Care Indicators (FCI) seven subscales: sources of play materials (source), varieties of play materials (variety), play activities (activity), household books (book), picture books (pbook), play materials (toy), and magazines and newspapers (magz). (ii) Bootstrap standard errors reported in column (2) are based on resampling with 1000 replications. (iii) The percentile 95% CI in column (3) uses usual sampling distribution cutoffs without bias correction; the BC 95% CI in column (4) corrects for a bias in the distribution of bootstrap estimates of standard errors; and the BCa 95% CI in column (5) corrects for bias and skewness in the distribution of bootstrap estimates of standard errors. (iv) *** *p* < 0.01, * *p* < 0.1.

**Table 5:** Estimates of indirect effects of parenting knowledge on language development through different parental investments.

Indirect Effect	Point Estimate	Bootstrap S. E.	95% CI (Percentile)	95% CI (BC)	95% CI (BCa)
	(1)	(2)	(3)	(4)	(5)
Knowledge on language through source	0.007 *	0.003	(0.001, 0.01)	(0.001, 0.02)	(0.001, 0.02)
Knowledge on language through variety	0.04 ***	0.01	(0.03, 0.06)	(0.03, 0.06)	(0.03, 0.06)
Knowledge on language through activity	0.03 ***	0.007	(0.01, 0.04)	(0.02, 0.05)	(0.02, 0.05)
Knowledge on language through book	0.01 **	0.004	(0.003, 0.02)	(0.002, 0.02)	(0.002, 0.02)
Knowledge on language through pbook	0.007 *	0.004	(0.001, 0.02)	(0.002, 0.02)	(0.002, 0.02)
Knowledge on language through toy	0.03 ***	0.01	(0.02, 0.05)	(0.02, 0.05)	(0.02, 0.05)
Knowledge on language through magz	0.004	0.004	(−0.002, 0.01)	(−0.002, 0.01)	(−0.002, 0.01)

Notes: (i) The mediators are the FCI seven subscales: sources of play materials (source), varieties of play materials (variety), play activities (activity), household books (book), picture books (pbook), play materials (toy), and magazines and newspapers (magz). (ii) Bootstrap standard errors reported in column (2) are based on resampling with 1000 replications. (iii) The percentile 95% CI in column (3) uses usual sampling distribution cutoffs without bias correction; the BC 95% CI in column (4) corrects for a bias in the distribution of bootstrap estimates of standard errors; and the BCa 95% CI in column (5) corrects for bias and skewness in the distribution of bootstrap estimates of standard errors. (iv) *** *p* < 0.01; ** *p* < 0.05; * *p* < 0.1.

**Table 6:** Estimates of indirect effects of parenting knowledge on motor development through different parental investments.

Indirect Effect	Point Estimate	Bootstrap S. E.	95% CI (Percentile)	95% CI (BC)	95% CI (BCa)
	(1)	(2)	(3)	(4)	(5)
Knowledge on motor through source	0.01 **	0.005	(0.003, 0.02)	(0.004, 0.02)	(0.004, 0.02)
Knowledge on motor through variety	0.06 ***	0.01	(0.04, 0.09)	(0.04, 0.09)	(0.04, 0.09)
Knowledge on motor through activity	0.03 ***	0.008	(0.01, 0.05)	(0.02, 0.06)	(0.02, 0.06)
Knowledge on motor through book	0.009 **	0.005	(0.001,0.02)	(0.002, 0.02)	(0.002, 0.02)
Knowledge on motor through pbook	0.01 **	0.005	(0.003, 0.02)	(0.003, 0.02)	(0.003, 0.02)
Knowledge on motor through toy	0.02 ***	0.006	(0.01, 0.04)	(0.01, 0.03)	(0.01, 0.03)
Knowledge on motor through magz	0.003	0.002	(−0.0008, 0.008)	(0.0001, 0.01)	(0.0001, 0.01)

Notes: (i) The mediators are the FCI seven subscales: sources of play materials (source), varieties of play materials (variety), play activities (activity), household books (book), picture books (pbook), play materials (toy), and magazines and newspapers (magz). (ii) Bootstrap standard errors reported in column (2) are based on resampling with 1000 replications. (iii) The percentile 95% CI in column (3) uses usual sampling distribution cutoffs without bias correction; the BC 95% CI in column (4) corrects for a bias in the distribution of bootstrap estimates of standard errors; and the BCa 95% CI in column (5) corrects for bias and skewness in the distribution of bootstrap estimates of standard errors. (iv) *** *p* < 0.01; ** *p* < 0.05.

**Table 7:** Estimates of indirect effects of parenting knowledge on social-emotional development through different parental investments.

Indirect Effect	Point Estimate	Bootstrap S. E.	95% CI (Percentile)	95% CI (BC)	95% CI (BCa)
	(1)	(2)	(3)	(4)	(5)
Knowledge on social-emotion through source	0.003	0.003	(−0.001, 0.009)	(0.0001, 0.02)	(0.0001, 0.02)
Knowledge on social-emotion through variety	0.03 ***	0.007	(0.01, 0.04)	(0.01, 0.04)	(0.01, 0.04)
Knowledge on social-emotion through activity	0.03 ***	0.006	(0.02, 0.04)	(0.02, 0.04)	(0.02, 0.04)
Knowledge on social-emotion through book	0.01 ***	0.005	(0.004, 0.02)	(0.004, 0.02)	(0.004, 0.02)
Knowledge on social-emotion through pbook	0.004	0.003	(−0.001, 0.01)	(0.0008, 0.02)	(0.0008, 0.02)
Knowledge on social-emotion through toy	0.02 **	0.007	(0.004, 0.03)	(0.003, 0.03)	(0.003, 0.03)
Knowledge on social-emotion through magz	0.002	0.002	(−0.001, 0.008)	(−0.001, 0.008)	(−0.001, 0.008)

Notes: (i) The mediators are the FCI seven subscales: sources of play materials (source), varieties of play materials (variety), play activities (activity), household books (book), picture books (pbook), play materials (toy), and magazines and newspapers (magz). (ii) Bootstrap standard errors reported in column (2) are based on resampling with 1000 replications. (iii) The percentile 95% CI in column (3) uses usual sampling distribution cutoffs without bias correction; the BC 95% CI in column (4) corrects for a bias in the distribution of bootstrap estimates of standard errors; and the BCa 95% CI in column (5) corrects for bias and skewness in the distribution of bootstrap estimates of standard errors. (iv) *** *p* < 0.01; ** *p* < 0.05.
